# Inflammasome lights up in systemic sclerosis

**DOI:** 10.1186/s13075-017-1420-z

**Published:** 2017-09-18

**Authors:** John Henderson, Steven O’Reilly

**Affiliations:** Immunology group, Faculty of Health and Life Sciences, Northumbria University, Ellison Building, Newcastle Upon Tyne, Tyne and Wear NE2 8ST UK

**Keywords:** Sclerosis, Fibrosis, Inflammasome, microRNA, Epigenetics, Interleukin-1, Caspase-1, MCC950

## Abstract

Systemic sclerosis (SSc) is a pro-fibrotic condition with a poorly understood aetiology. Evidence presented by Artlett et al. in this issue suggests that the microRNA miR-155 is key, with its involvement dependent on the NLRP3 inflammasome. This links epigenetic events with inflammasome signalling in SSc and opens the door to new therapeutic strategies for the treatment of SSc.

## Main text

Systemic sclerosis (SSc) is an idiopathic autoimmune disease characterised by inflammation, vascular problems, cytokine dysregulation and ultimately fibrosis, which accounts for the poor prognosis and eventual mortality. At present, treatment of SSc is limited by incomplete understanding of pathogenesis and although immunosupressive strategies are beneficial for lung and skin, the benefits are often modest. Excessive collagen production by over-activated fibroblasts is the key driver of the fibrosis, with a spike in the innate immune response and, in particular, Toll-like receptor (TLR) signalling recently identified as responsible for the fibroblast activation.

Since its discovery, the NLR family pyrin domain containing 3 (NLRP3) inflammasome has become increasingly implicated in numerous inflammatory conditions, raising the suspicion that it has a key role in SSc. This suspicion has been confirmed by Artlett et al. [1], who present a compelling case for the NLRP3 inflammasomme-mediated importance of miR-155 (microRNA-155) in SSc, an outcome that carries high clinical significance. They show that NLRP3-deficient fibroblasts and fibroblasts treated with the caspase-1 inhibitor YVAD potently reduced miR-155 expression and decreased collagen synthesis, with the addition of SSc-promoting stimuli having no effect—thus, it appears the inflammasome regulates miR-155 and collagen levels. It is suggested that the interplay between the innate immune system and microRNA expression and the subsequent activation of fibroblasts have a dominant pathogeneic role in SSc.

In recent years small non-coding microRNAs have emerged as key players in a number of diseases [2], with SSc apparently now added to their number. Widely regarded as the ‘fine tuners’ of gene expression, a single microRNA can have hundreds of targets. Artlett et al. [1] show that miR-155 is elevated in SSc dermal and lung fibroblasts and that deletion of miR-155 in mouse cells treated with bleomycin (a model for scleroderma) reverts collagen synthesis back to basal levels. They then performed an elegant experiment whereby re-introducing miR-155 back into the knockout cells via a retroviral vector caused an upregulation of collagen which was augmented in the presence of bleomycin—outlining the importance of miR-155 in SSc. This is underscored by the fact that, despite the transfection efficacy of the cells to take up the vector being only 10% on average, a dramatic increase in collagen was still seen.

From a therapeutic perspective this central importance of miR-155 is very exciting given a miR-155 inhibitor has recently been shown to effectively treat dermal fibrosis in a mouse model [3], with these results suggesting that, if applied intravenously, it could have a similar effect against SSc fibrosis.

Mechanistically, however, the big question of how enhanced miR-155 drives collagen synthesis remains unanswered. We too have seen elevated miR-155 levels in SSc patients in association with increased collagen, but the targets of miR-155 that mediate this increase are still under review, with inhibitors of collagen regulation appearing likely candidates. Artlett et al. [1] show a dependence of mir-155 on downstream IL-1 signalling, with the IL-1 receptor antagonist (IL-1RA) shown to block increased collagen synthesis in the presence of bleomycin or the miR-155 vector. These findings are substantiated by Abtahi et al. [4], who found that single nucleotide polymorphisms (SNPs) in the IL-1 gene cluster may affect SSc susceptibility. This suggests that using IL-1 receptor antagonists may be useful in SSc. Indeed a clinical trial is currently underway to determine the efficacy of using an IL-1 receptor blocker in SSc, with results eagerly anticipated given such drugs are highly efficient in IL-1-driven diseases such as hereditary systemic inflammatory disorders and gout.

Artlett et al. [1] expand on this by going on to propose a feed-forward mechanism whereby IL-1 upregulates miR-155, which itself interacts with the inflammasome to promote IL-1 activation, providing a feasible explanation for how chronic collagen over-expression could occur. The authors don’t identify any specific targets of miR-155 prior to inflammasone upregulation within this mechanism and the molecules being repressed remain to be elucidated. miR-155 is induced by TLR stimulation and is used to negatively regulate TLR signalling. The authors demonstrate that IL-1β induces miR-155 expression, which may be a mechanism of negative feedback. Taken together, these results raise the possibility of the inflammasome functioning as a nexus for the currently unknown triggers leading to SSc pathogenesis. This fits with the NLRP3 inflammasone’s sensitivity to environmental stimuli such as toxins and drugs, which it has been speculated may be the triggers for SSc. Consequently, enhanced NLRP3 activity and IL-1β production in response to an unknown external insult may be what fuels the disease progression.

Finally, Artlett et al [1] show the dependence of the notorious master regulator of fibrosis, transforming growth factor-β1 (TGF-β1), on miR-155, with bleomycin unable to induce TGF-β1 in miR-155 knockout fibroblasts. This further emphasises that miR-155 is a key proponent of the fibrosis observed in SSc and may also play a key role in other pro-fibrotic diseases where TGF-β1 is involved. Quite how miR-155 regulates TGF-β1 remains unknown and is likely to be an indirect mechanism as no seed region in the 3′ UTR is predicted to bind miR-155.

One way in which this work could be taken forward would be to replicate the experiments performed in this study in vivo, using the same mouse models. Additionally, the transgenic miR-155 overexpressing mouse model Rm155LG could be utilised to verify that enhanced miR-155 expression increases collagen production and fibrosis susceptibility in vivo.

Most importantly of all, the dependence of miR-155-driven effects specifically on NLRP3 inflammasome activity also opens up new therapeutic avenues, with the recently developed small molecule inhibitor of the NLRP3 complex MCC950 touted as an excellent candidate for treating NLRP3-related pathologies [5], a theory that could be probed by testing MCC950 on the previously mentioned models of SSc. MCC950 inhibits NLRP3 in response to canonical and non-canonical ligands, which is important as the ligands for activation of NLRP3 in SSc are not yet known. In liver fibrosis it has been demonstrated that NLRP3 activation occurs in response to palmitic acid and the transformation of the quiescent hepatic cell to a hepatic stellate cell [6]; hence, we postulate that similar ligands could be activating the inflammasome in SSc.

## Abbreviations

UTR: Untranslated region
IL-1β: Interleukin-1β
IL-RA: IL-1 receptor antagonist
miR-155: MicroRNA-155
NLRP3: NLR pyrin domain containing 3
SNP: Single nucleotide polymorphism
SSc: Systemic sclerosis
TGF-β1: Transforming growth factor-β1
TLR: Toll-like receptor

## References

[1] Artlett CM, Sassi-Gaha S, Hope JL, Feghali-Bostwick CA, Katsikis PD (2017). Mir-155 is overexpressed in systemic sclerosis fibroblasts and is required for NLRP3 inflammasome-mediated collagen synthesis during fibrosis. Arthritis Res Ther.

[2] O’Reilly S (2016). MicroRNAs in fibrosis: opportunities and challenges. Arthritis Res Ther.

[3] Yan Q, Chen J, Li W, Bao C, Fu Q (2016). Targeting miR-155 to treat experimental scleroderma. Sci Rep.

[4] Abtahi S, Farazmand A, Mahmoudi M, Ashraf-Ganjouei A, Javinani A, Nazari B (2015). IL-1A rs1800587, IL-1B rs1143634 and IL-1R1 rs2234650 polymorphisms in Iranian patients with systemic sclerosis. Int J Immunogenet.

[5] Coll RC, Robertson AAB, Chae JJ (2015). A small molecule inhibitor of the NLRP3 inflammasome is a potential therapeutic for inflammatory diseases. Nat Med.

[6] Duan NN, Liu XJ, Wu J (2017). Palmitic acid elicits hepatic stellate cell activation through inflammasomes and hedgehog signaling. Life Sci.

